# Origin of fundus hyperautofluorescent spots and their role in retinal degeneration in a mouse model of Goldmann-Favre syndrome

**DOI:** 10.1242/dmm.012112

**Published:** 2013-07-04

**Authors:** Nan-Kai Wang, Chi-Chun Lai, Chi-Hsiu Liu, Lung-Kun Yeh, Chai Lin Chou, Jian Kong, Takayuki Nagasaki, Stephen H. Tsang, Chung-Liang Chien

**Affiliations:** 1 Department of Anatomy and Cell Biology, National Taiwan University, Taipei 100, Taiwan; 2 Department of Ophthalmology, Chang Gung Memorial Hospital, Linkou, Taoyuan 333, Taiwan; 3 Chang Gung University College of Medicine, Linkou, Taoyuan 333, Taiwan; 4 Faculty of Medicine, The University of British Columbia, Vancouver, BC V6T 1Z4 Canada; 5 Bernard and Shirlee Brown Glaucoma Laboratory, Department of Pathology and Cell Biology, Columbia University, New York, NY 10032, USA; 6 Edward S. Harkness Eye Institute, Columbia University, New York, NY 10032, USA

## Abstract

Goldmann-Favre syndrome, also known as enhanced S-cone syndrome, is an inherited retinal degeneration disease in which a gain of photoreceptor cell types results in retinal dysplasia and degeneration. Although microglia have been implicated in the pathogenesis of many neurodegenerative diseases, the fundamental role of these cells in this disease is unknown. In the current study, sequential analyses suggest that microglia are recruited and appear after outer nuclear layer folding. By crossing *rd7* mice (a model for hereditary retinal degeneration owing to *Nr2e3* mutation) with mice carrying the macrophage Fas-induced apoptosis (Mafia) transgene, we generated double-mutant mice and studied the role of the resident retinal microglia. Microglial cells in these double-mutant mice express enhanced green fluorescent protein (EGFP) and a suicide gene that can trigger Fas-mediated apoptosis via systemic treatment with AP20187 (FK506 dimerizer). We demonstrated that more than 80% of the EGFP+ cells in retinas from *rd7/rd7;Tg/Tg* mice express Iba-1 (a microglial marker), and resident microglia are still present in the retina because AP20187 does not cross the blood-brain barrier. Hence, only circulating bone marrow (BM)-derived microglia are depleted. Depletion of circulating BM-derived microglia accelerates retinal degeneration in *rd7* mice. An increased number of autofluorescent (AF) spots is a consequence of resident microglia proliferation, which in turn establishes an inflammatory cytokine milieu via the upregulation of *IL-1β*, *IL-6* and *TNFα* expression. This inflammation is likely to accelerate retinal degeneration. This study not only identifies inflammation as a crucial step in the pathogenesis of retinal degeneration, but also highlights the involvement of specific cytokine genes that could serve as future treatment targets in retinal degenerations.

## INTRODUCTION

Retinal degeneration in *rd7* mice is caused by a spontaneous mutation in the *Nr2e3* gene. In addition, this mouse strain is a model for Goldmann-Favre syndrome [also known as enhanced S-cone syndrome (ESCS); OMIM 268100 (http://omim.org/entry/268100)] (Akhmedov et al., 2000). In these mice, a gain of photoreceptor cell types results in retinal dysplasia and degeneration.

Recently, we described newly identified characteristics – including diffuse retinal white dots, hyperautofluorescent (hyper-AF) spots and retinal rosettes – in a 6-year-old boy with ESCS who carried a homozygous R311Q mutation in the *NR2E3* gene (Wang et al., 2009). His phenotypic manifestations were similar to those of ‘young’ *rd7* mice. We demonstrated that F4/80-positive microglia, rather than retinal pigment epithelium (RPE) cells, contributed to these AF spots. Most of these cells were present inside retinal rosettes and presumably helped RPE cells phagocytose this outer segment (OS) debris within the rosettes. Although these data demonstrated the presence of comparable retinal characteristics in human ESCS and a mouse model of the disease, the fundamental role of microglia in retinal degeneration is unknown.

Microglia, which are part of the mononuclear phagocytic system, act as the first and main form of active immune defense in the central nervous system (CNS), including in the retina (Kreutzberg, 1996; Cuadros and Navascués, 1998; Hanisch and Kettenmann, 2007; Tambuyzer et al., 2009). Microglial activation is characterized by the expression of various microglial and/or macrophagic markers. In the retina, microglial activation has been demonstrated in injury (Ng and Streilein, 2001; Langmann, 2007; Joly et al., 2009), ischemia (Zhang et al., 2005; Ritter et al., 2006; Sivakumar et al., 2011) and degeneration (Langmann, 2007; Sasahara et al., 2008; Arroba et al., 2011). Microglial cells from two origins exist in the retina: resident microglia and circulating bone marrow (BM)-derived microglia, with the former entering from hyaloid vessels and being thought to be associated with neuronal death in retinal histogenesis (Ashwell et al., 1989), whereas the latter enter from the optic nerve after retinal vascularization (Caicedo et al., 2005; Hou et al., 2006). Although BM transplantation approaches have the potential to systemically remove macrophages in order to study their function *in vivo* in normal or disease models, pre-BM-transplantation irradiation damages resident microglia, which might change the immune environment of the retina (Amoakul et al., 1992; Kaneko et al., 2008).

Burnett and colleagues generated mice that carry the transgene for macrophage Fas-induced apoptosis (Mafia) (Burnett et al., 2004; Burnett et al., 2006). This transgene (Tg: *Csf1r-EGFP-NGFR/FKBP1A/TNFRSF6*) is under the control of the *c-fms* promoter, which drives the expression of the CSF-1 receptor in cells of the mononuclear phagocytic system, including monocytes, macrophages, dendritic cells (DC), Kupffer cells, Langerhans cells, osteoclasts and microglial cells (Cecchini et al., 1994). In Mafia mice, cells of the macrophage lineage express the EGFP and a membrane-bound suicide protein that can be activated by the covalently linked dimerizing reagent AP20187. Henceforth, we will use ‘Tg/Tg’ to refer to mice that are homozygous for this transgene.

**TRANSLATIONAL IMPACT**

**Clinical issue**

Goldmann-Favre syndrome, also known as enhanced S-cone syndrome, is an inherited eye disorder characterized by retinal degeneration. Previously, this group reported the appearance of diffuse retinal white dots, hyperautofluorescent spots and retinal rosettes in young patients with Goldmann-Favre syndrome, and they recently showed that these features are also seen in young *rd7* (retinal degeneration) mice. Retinal microglial cells, of which there are two origins – resident microglia and circulating bone marrow (BM)-derived microglia – have been implicated in the pathogenesis of Goldmann-Favre syndrome and it was suggested that microglial cells contribute to the development of hyperautofluorescent spots. However, the fundamental role of microglial cells in Goldmann-Favre syndrome pathogenesis is unknown.

**Results**

Here, the authors exploited Mafia (macrophage Fas-induced apoptosis) transgenic mice to explore the contribution of microglial cells to Goldmann-Favre syndrome. Temporal control of a circulating BM-derived microglia population was achieved by introducing the Mafia transgene into the *rd7* mouse model of Goldmann-Favre syndrome. Systemic ablation of BM-derived microglia can be induced by treatment with a synthetic dimerizer, AP20187. Because this compound does not cross the blood-brain barrier, resident retinal microglia are spared but circulating BM-derived microglia are ablated after AP20187 administration. After systemic depletion of circulating BM-derived microglia in young mice, characteristics that mimicked those of later stages of retinal degeneration in *rd7* mice were observed in cilioretinal flatmounts. Moreover, analyses of the outer nuclear layer revealed a decreased nuclei count in photoreceptors. Inflammatory cytokines IL-1β, IL-6, TNFα and monocyte chemotactic protein (MCP-1) were upregulated, whereas the anti-inflammatory cytokine TGF-1 was found to be downregulated in the retina.

**Implications and future directions**

These data suggest that circulating BM-derived microglia have a role in suppressing intraocular inflammation. Loss of the microglial population can thereby enhance inflammatory pathways and accelerate retinal degeneration. This study highlights the opposing roles of resident microglia, which are still present in the retina of mutant mice, and circulating BM-derived microglia in retinal degeneration. Future studies dedicated to the search for therapeutic agents that intervene in the inflammatory processes involved could provide a novel treatment strategy for inherited retinal degeneration and other diseases characterized by degeneration of neurons, such as Alzheimer’s disease.

In the current study, we took advantage of Mafia transgenic mice to mark the origin of AF spots with the EGFP reporter in *rd7* mice.

## RESULTS

### Spatial and temporal distribution of AF spots and rosettes in *rd7* mice

**Fig. 1.**
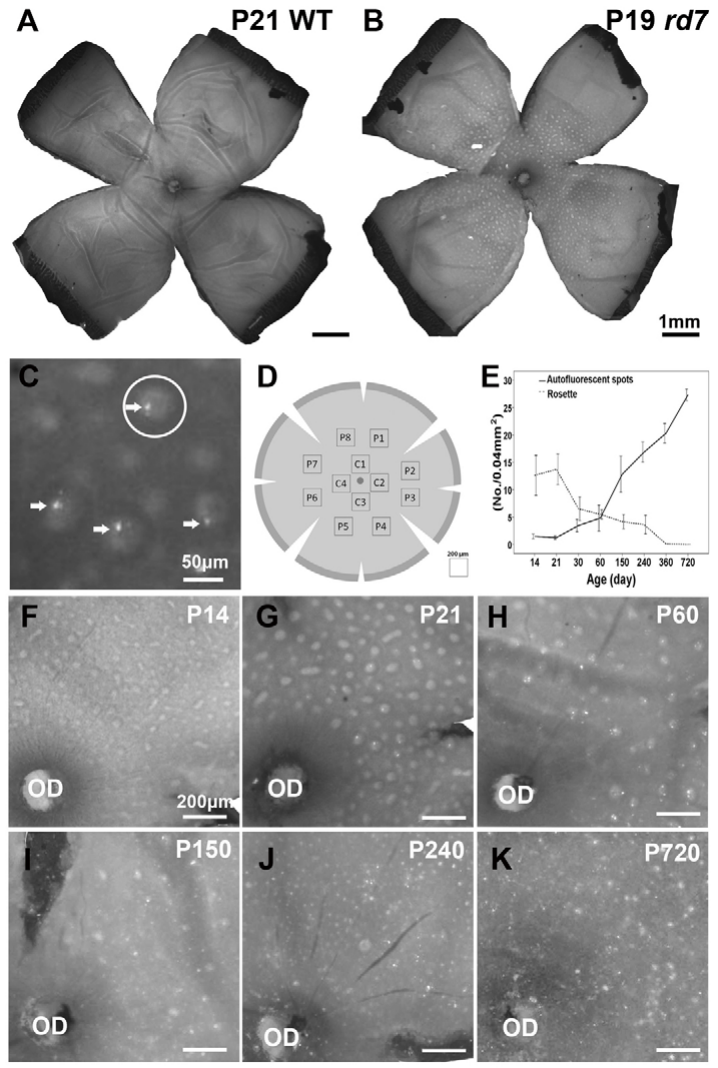
**Spatial and temporal distribution of autofluorescent (AF) spots and rosettes in *rd7* mice.** (A,B) Cilioretinal flatmount of P21 wild-type (WT; A) and P19 *rd7* (B) mice. (C) Higher magnification of the boxed area from panel B shows examples of AF spots (arrows) and rosettes (circular halos; an example is circled). Note that the AF spots were located inside the rosettes. (D) Scheme of a cilioretinal flatmount showing the areas in which the AF spots and rosettes were counted. C, central; P, peripheral. (E) The number of AF spots and rosettes in the cilioretinal flatmount (mean ± s.d.) was determined at P14 (*n*=4), P21 (*n*=3), P30 (*n*=3), P60 (*n*=4), P150 (*n*=3), P240 (*n*=3), P360 (*n*=2) and P720 (*n*=2). (F) By P14, rosettes were distributed throughout the cilioretinal flatmount. Note that AF spots were absent. (G) By P21, some AF spots were located inside rosettes; the AF spots were not distributed along the retinal vessels. (G-K and E) The number of rosettes decreased gradually, whereas the number of AF spots increased. OD, optic disc.

Our previous study showed that retinal rosettes corresponded to retinal folds, and that AF spots corresponded to microglial cells located between the neural retina and the RPE (Wang et al., 2009). To investigate the relationship between autofluorescence, microglial infiltration and retinal degeneration, we counted the AF spots and rosettes in the cilioretinal flatmounts of *rd7* mice between postnatal day 14 (P14) and P720 (Fig. 1). Fig. 1A,B show the cilioretinal flatmounts of P21 wild-type (WT; A) and P19 *rd7* (B) mice. Fig. 1C shows examples of AF spots (arrows) and rosettes (circular halos). Although the number of retinal rosettes increased from P14 to P21, the number of rosettes decreased progressively from P21 through to P720. In contrast, AF spots that were absent at P14 were first detected as early as P21 (Fig. 1G), and the number increased gradually with age (Fig. 1F–K). We found that the AF spots were not distributed along the retinal vessels, but rather were correlated with rosettes between P21 and P60.

### Abnormal accumulation of material in the OS of *rd7* mice

**Fig. 2.**
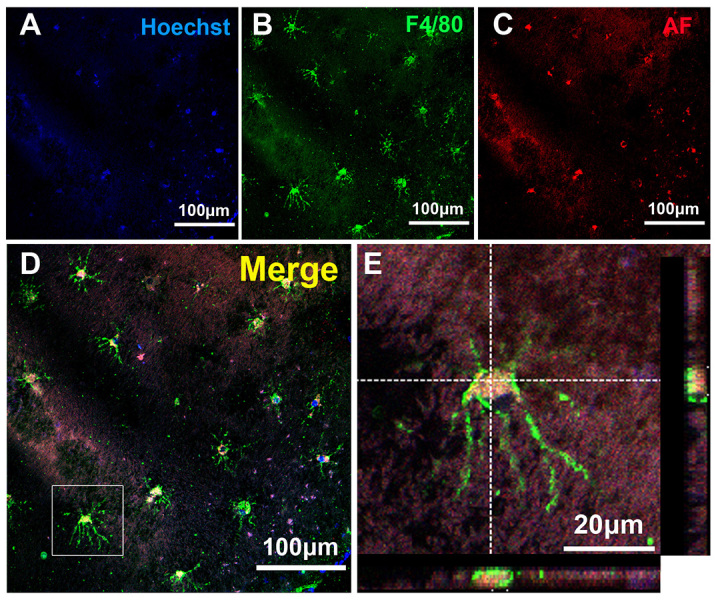
**Immunohistochemistry of the cilioretinal flatmounts from 2-month-old *rd7* mouse retinas using anti-F4/80 antibodies.** (A–D) Confocal images taken from the cilioretinal flatmounts showed the presence of several ramified microglia ‘lying’ above the RPE layer. These cells were colocalized with F4/80 (green) and autofluorescent material (red). (E) Crosshairs and high-magnification viewing of single cells located inside the boxed area from panel D show the presence of autofluorescent material within the cytoplasm of an F4/80-positive cell. F4/80, microglial marker; AF, autofluorescence.

Previously, abnormal accumulation of material at the photoreceptor-RPE interface was documented in *Nrl*^−/–^ mice, another mouse model of ESCS (Mustafi et al., 2011). In addition, in our previous study, we found cells that were stained positively for F4/80 inside the retinal rosettes (Wang et al., 2009). To characterize these cells further, we performed an immunochemical analysis of retinas in *rd7* mice. Confocal images taken from the cilioretinal flatmounts of 2-month-old *rd7* mouse retinas probed with anti-F4/80 antibodies revealed the presence of ramified microglia ‘lying’ above the RPE layer (Fig. 2). These cells were colocalized with F4/80 (green, Fig. 2B) and autofluorescent material (red, Fig. 2C). Crosshairs and high-magnification viewing revealed the presence of autofluorescent material within the cytoplasm of an F4/80-positive cell (Fig. 2E).

**Fig. 3.**
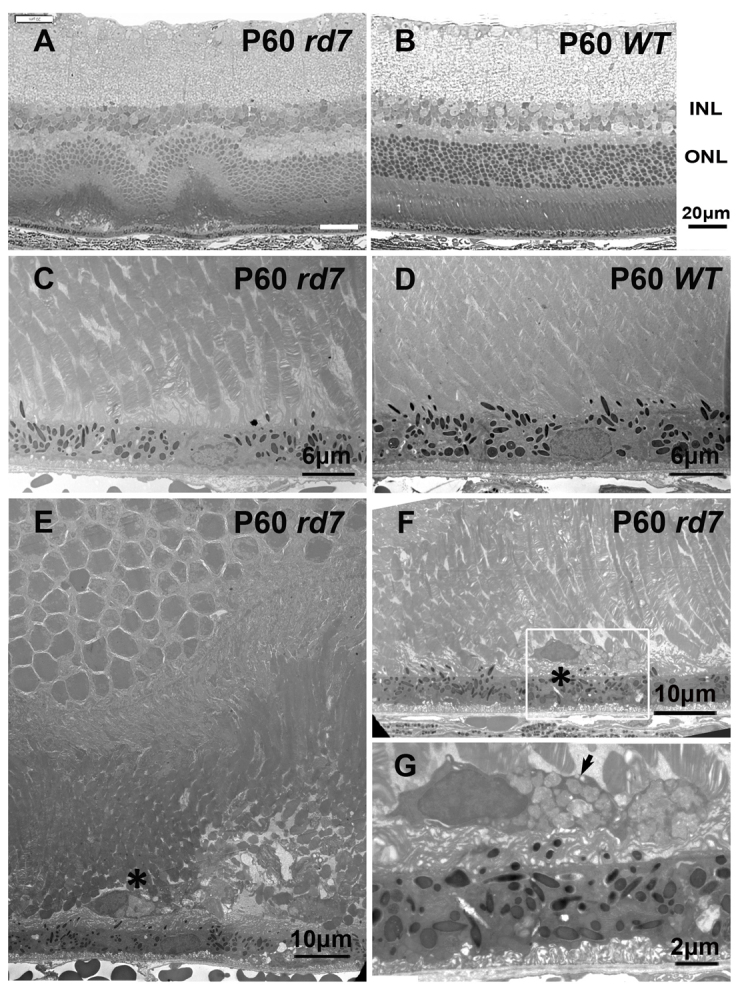
**Histology and electron microscopy of P60 *rd7* mouse retinas compared with P60 wild-type mouse retinas.** (A) Semi-thin section of an *rd7* mouse retina showing outer nuclear layer foldings. (B) Control: semi-thin section of a WT mouse retina. INL, inner nuclear layer; ONL, outer nuclear layer. (C,D) Ultrathin section of retinas of *rd7* and WT mice, showing the junction between the outer segment (OS) and RPE. (E,F) The ultrastructural analysis of the retina of *rd7* mice revealed the presence of microglial cells between the OS and the RPE. Asterisks indicate microglia. (G) Higher magnification of the boxed area from panel F, showing the accumulation of lysosomes inside a microglial cell (arrow).

To characterize the autofluorescent material further, we performed a histological analysis of *rd7* and WT (C57BL/6) mouse photoreceptors. Abnormal folding at the outer nuclear layer (ONL), OS and inner segment was noted in P60 *rd7* mice (Fig. 3A), whereas this folding was not found in P60 WT mouse retinas (Fig. 3B). To confirm this finding, we examined the retinas of *rd7* and WT mice by using transmission electron microscopy (TEM) (Fig. 3C–H). In *rd7* mouse retinas, some microglia (different to RPE cells) were detected between the OS and the RPE (asterisks in Fig. 3E,F). A higher-magnification view of the TEM images showed the accumulation of lysosomes inside microglial cells (arrow in Fig. 3G), and microglial cells were more likely to be found under retinal rosettes (Fig. 3E,F). No microglia were found in WT mouse retinas (Fig. 3D).

### Distribution of microglia in *rd7/rd7;Tg/Tg* mice

**Fig. 4.**
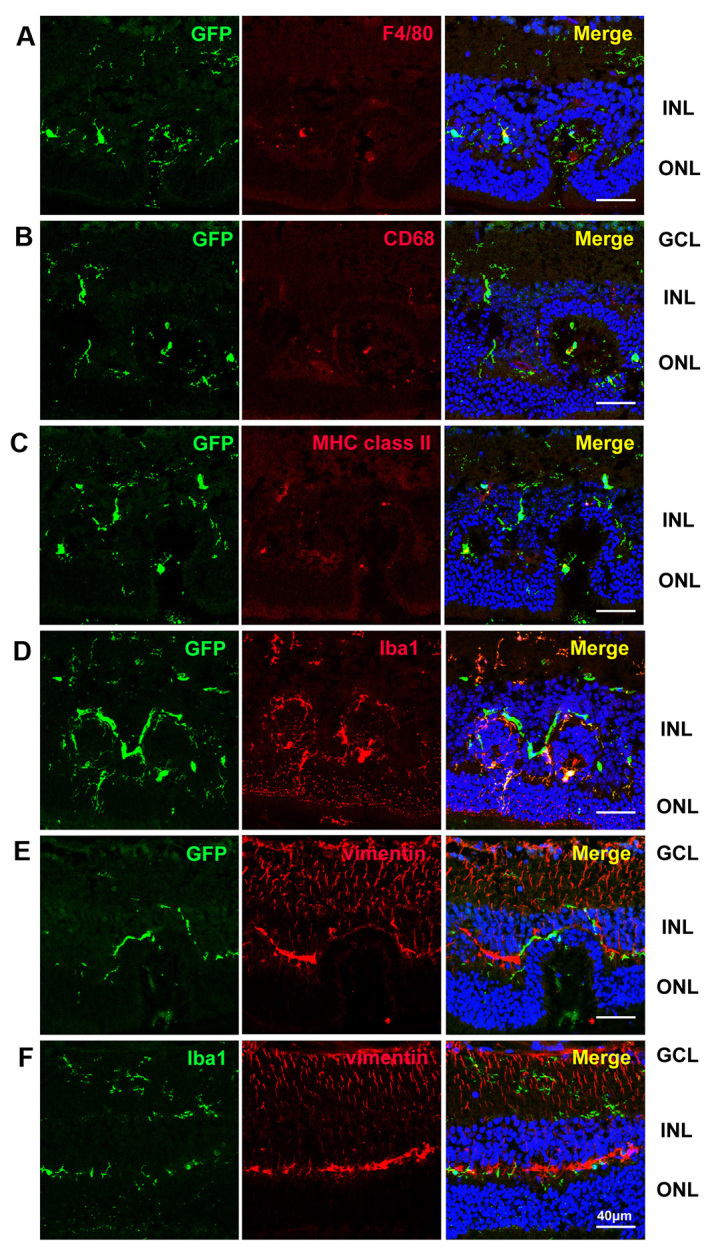
**Confocal images showing the distribution of EGFP, F4/80, CD68, MHC-II, Iba-1 and vimentin labeling in the retina of a 3-month-old *rd7/rd7;Tg/Tg* mouse.** Of the EGFP+ cells, 45.4±9.5% expressed F4/80 (A), 47.7±10.4% expressed CD68 (B), 39.7±7.7% expressed MHC class II (C) and 84.2±8.0% expressed Iba-1 (D). Less than 10% of the EGFP+ cells expressed vimentin (E). GCL, ganglion cell layer; INL, inner nuclear layer; ONL, outer nuclear layer.

To investigate the role of circulating BM-derived microglia in *rd7* mice, we generated *rd7/rd7;Tg/Tg* double homozygotes via a two-generation outcross-intercross series. Theoretically, the transgene is under the control of the *c-fms* promoter and is expressed in macrophages, monocytes, microglia and DCs (Burnett et al., 2004; Burnett et al., 2006). Previously, microglia were classified into various subtypes (Zhang et al., 2005). To investigate further whether EGFP was expressed in these microglia and whether EGFP expression colocalized with microglial markers, we performed an immunohistochemical analysis of the retinas of *rd7/rd7;Tg/Tg* mice. Among the EGFP+ cells, 45.4±9.5% expressed F4/80, 47.7±10.4% expressed CD68, 84.2±8.0% expressed Iba-1 and 39.7±7.7% expressed MHC class II (MHC-II). Less than 10% of the EGFP+ cells expressed vimentin. We also found that the EGFP+ cells were distributed in the ganglion cell layer (GCL), inner plexiform layer (IPL), inner nuclear layer (INL), outer plexiform layer (OPL), and between the OS and the RPE (Fig. 4). In summary, the majority of EGFP+ cells (>80%) in *rd7/rd7;Tg/Tg* mice expressed Iba-1, and few EGFP+ cells were labeled by vimentin.

### Circulating BM-derived microglia deficiency in *rd7* mice

We introduced inducible circulating BM-derived microglia deficiency into the mouse model of ESCS by crossbreeding *rd7* onto the Mafia mice. The *rd7/rd7;Tg/Tg* mice had similar physical characteristics as those of *rd7* mice and survived more than 1 year, when allowed. The growth of *rd7/rd7;Tg/Tg* mice was similar to that of *rd7* mice. However, after circulating BM-derived microglia ablation using AP20187, the body weight in the AP20187 mice did not increase as fast as in the vehicle-treated animals (supplementary material Fig. S1).

To investigate whether AP20187 can systemically deplete circulating BM-derived microglia in *rd7/rd7;Tg/Tg* mice, we injected AP20187 intravenously at a dose of 10 mg/kg of body weight for 5 consecutive days, and then twice weekly at a reduced dose of 1 mg/kg of body weight. We found a marked decrease in EGFP expression in peritoneal cells, suggesting that AP20187 can deplete circulating BM-derived microglia in *rd7/rd7;Tg/Tg* mice (supplementary material Fig. S2A). An enlarged spleen was noted in *rd7/rd7;Tg/Tg* mice injected with AP20187 compared with the vehicle-injected mice (supplementary material Fig. S2G,D). We stained apoptotic cells selectively in the spleen section using an anti-active-caspase-3 antibody and TUNEL, and found a marked increase in apoptotic cells in AP20187-injected *rd7/rd7;Tg/Tg* mice (supplementary material Fig. S2B,C,E,F). Our hematoxylin and eosin analysis demonstrated an increase in the nucleus:cytoplasm ratio and loss of red pulp in the spleen (data not shown), suggesting the presence of extramedullary hematopoiesis in *rd7/rd7;Tg/Tg* mice after long-term depletion of circulating BM-derived microglia.

### Acceleration of retinal degeneration in *rd7/rd7;Tg/Tg* mice after systemic depletion of circulating BM-derived microglia

**Fig. 5.**
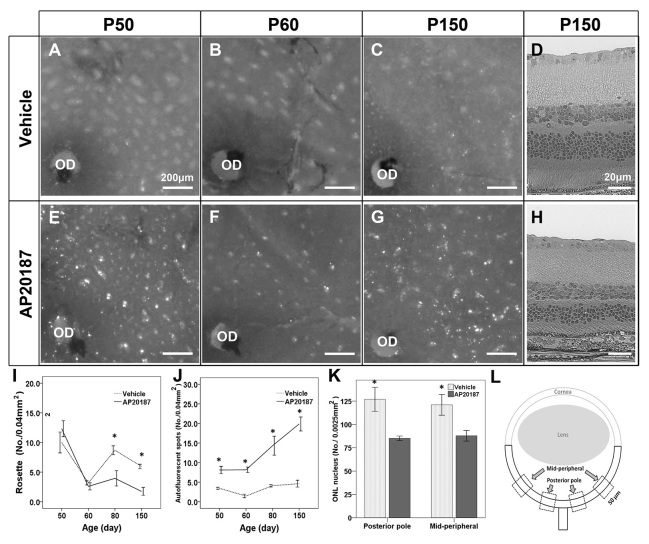
**Accelerated retinal degeneration after systemic depletion of circulating BM-derived microglia in *rd7/rd7;Tg/Tg* mice.** (A–D) The cilioretinal flatmounts and histology from *rd7/rd7;Tg/Tg* mice treated with vehicle at P50 (A), P60 (B) and P150 (C,D). (E–H) The cilioretinal flatmounts and histology from *rd7/rd7;Tg/Tg* mice treated with AP20187 at P50 (E), P60 (F) and P150 (G,H). (I) The number of rosettes in the cilioretinal flatmounts (mean ± s.d.) was determined at P50 (*n*=8), P60 (*n*=11), P80 (*n*=3) and P150 (*n*=4). (J) The number of AF spots in the cilioretinal flatmounts (mean ± s.d.) was determined at P50 (*n*=8), P60 (*n*=11), P80 (*n*=3) and P150 (*n*=4). (K) The number of nuclei in the outer nuclear layer (ONL) was significantly decreased in mice injected with AP20187 for 5 months compared with mice injected with vehicle. (L) Scheme of an eye section, showing the areas in which the ONL nuclei were counted. OD, optic disc. *P<0.05.

To confirm the role of circulating BM-derived microglia in *rd7/rd7;Tg/Tg* mice, we investigated the morphological changes in the retinal rosettes and AF spots after AP20187 injection (Fig. 5A–G). We found a marked and rapid decrease in the number of retinal rosettes, which was accompanied by an increase in the number of AF spots (Fig. 5I,J). As mentioned above, increasing numbers of AF spots and decreased numbers of rosettes resemble the characteristics found in older *rd7* mice, suggesting an acceleration of retinal degeneration. For this reason, we counted the number of nuclei in the photoreceptors of *rd7/rd7;Tg/Tg* mice after 5 months of AP20187 injections, as well as that for the vehicle-injected mice (Fig. 5L). There was a significant decrease in the number of nuclei in the ONL in *rd7/rd7;Tg/Tg* mice injected with AP20187 compared with vehicle-injected mice (posterior pole, *P*=0.022; midperipheral area, *P*=0.031; *n*=3) (Fig. 5D,H,K). These data suggest that the systemic depletion of circulating BM-derived microglia accelerates retinal degeneration in *rd7/rd7;Tg/Tg* mice.

### Changes in the number of proliferative cells after systemic depletion of circulating BM-derived microglia in *rd7/rd7;Tg/Tg* mice

**Fig. 6.**
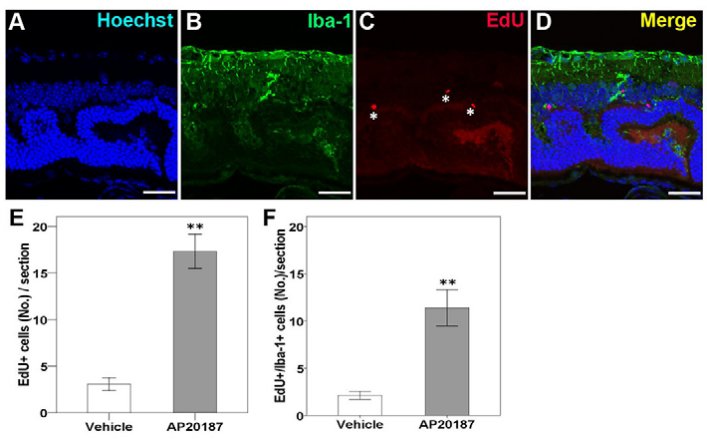
**Confocal images showing proliferative resident microglia in the retina of *rd7/rd7;tg/tg* mice after 4 weeks of injection of AP20187.** (A–D) Proliferative cells located in the outer plexiform layer (OPL), outer nuclear layer (ONL) or ganglion cell layer exhibited EdU labeling (asterisks in C). Colocalized labeling of EdU and Iba-1 was detected (D). (E,F) The number of EdU-labeled cells was significantly increased in mice injected with AP20187 for 1 month compared with mice injected with vehicle (*n*=3 in each group). The majority of the EdU-labeled cells were colocalized with Iba-1 (F). Asterisks indicate significant difference: **P<0.001. Scale bars: 30 μm.

To investigate whether retinal resident microglia proliferation accounts for the significantly increased number of AF spots observed in *rd7/rd7;Tg/Tg* mice injected with AP20187, we performed EdU labeling and Iba-1 immunostaining in *rd7/rd7;Tg/Tg* mice 1 month after treatment with AP20187 or vehicle. Compared with retinas from vehicle-injected *rd7/rd7;Tg/Tg* mice, there were significantly more labeled dividing resident microglial cells in retinas from AP20187-injected mice (Fig. 6), demonstrating an essential role for the resident microglia after the systemic depletion of circulating BM-derived microglia.

### Changes in the expression of retinal cytokines/chemokines after systemic depletion of circulating BM-derived microglia in *rd7/rd7;Tg/Tg* mice

**Fig. 7.**
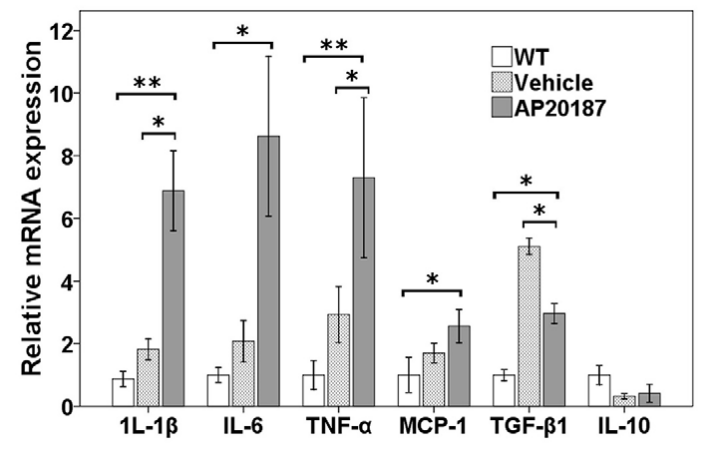
**Relative gene expression after systemic depletion of circulating BM-derived microglia.** Quantification of gene expression changes by qRT-PCR at P60 (4 weeks after being treated with AP20187 or vehicle; *n*=3 in each group), expressed as mean fold change in comparison with wild type (WT) ± 1 s.d., showing a trend of downregulation of the gene encoding the anti-inflammatory cytokine TGFβ1, and upregulation of genes encoding the inflammatory cytokines IL-1β, IL-6, TNFα and monocyte chemotactic protein (MCP-1). Asterisks indicate significant difference: *P<0.05, **P<0.001.

Next, we investigated the mechanism underlying the retinal degeneration observed after circulating BM-derived microglia depletion. Retinas from WT mice (C57BL/6), *rd7/rd7;Tg/Tg* mice treated with AP20187, and *rd7/rd7;Tg/Tg* mice treated with vehicle were extracted using lysis buffer and were subjected to quantitative real-time PCR (qRT-PCR; *n*=3 in each group). The genes that encode the inflammatory cytokines interleukin-1β (IL-1β), IL-6, tumor necrosis factor-α (TNFα) and monocyte chemotactic protein (MCP-1) were upregulated after systemic depletion of circulating BM-derived microglia; however, the gene that encodes the anti-inflammatory cytokine TGFβ1 was downregulated (Fig. 7). TNFα was chosen for its role as the classical activator and initiator of cytotoxicity, as well as a sustainer of macrophage responses. We thus concluded that resident microglia play an essential role in, and that their proliferation upregulates, the expression of the *IL-1β*, *IL-6* and *TNFα* genes in the retina, after circulating BM-derived microglia depletion.

## DISCUSSION

Microglia have been reported to play a central role in chronic degenerative conditions of the CNS, including Alzheimer’s and Parkinson’s diseases, and amyotrophic lateral sclerosis (Block et al., 2007; Gentleman, 2013). In the present study, we reported the opposing roles of resident microglia and circulating BM-derived microglia in retinal degeneration.

### Sequential appearance of AF spots, rosettes and microglial cells

In a previous study, we found microglia inside the ONL folding, which appeared as white dots in fundoscopy and retinal rosettes in cilioretinal flatmounts (Wang et al., 2009). In the current study, TEM and immunochemical analyses demonstrated the presence of lysosomes inside these microglia, which appeared as AF spots in the cilioretinal flatmounts. Sequential analyses of the retinal rosettes and AF spots indicated that the retinal rosettes appeared earlier than the AF spots, suggesting that microglia are recruited and appear after ONL folding. The increased number of AF spots found in the older *rd7* mice might be related to an increase in activated-lysosome-laden microglia, which represent a later stage of retinal degeneration.

Based on the spatial and temporal distribution of the AF spots and rosettes, we found that microglial cells are not simply bystanders in *rd7* mice, but might contribute to the degenerative process. We hypothesize that microglia play a positive role in maintaining the environment of photoreceptors by initially cleaning up debris between photoreceptors and the RPE. A similar symbiosis between microglia and photoreceptors was found in other studies (Banerjee and Lund, 1992; Ritter et al., 2006; Sasahara et al., 2008; Joly et al., 2009; Arroba et al., 2011). Another study demonstrated that BM-derived cells can eliminate amyloid deposits in Alzheimer’s disease via a cell-specific phagocytic mechanism (Simard et al., 2006).

### Advantages of Mafia mice compared with BM chimeric mice

An advantage of our murine model of circulating BM-derived microglia deficiency in retinal degeneration is that, in contrast to most published macrophage-deficiency models, on which BM transplantations were performed after systemic irradiation, in our model, the resident microglia are not ablated after systemic depletion of BM-derived microglia. This helped us investigate the role of resident and circulating BM-derived microglia in retinal degeneration. Although many researchers have used BM transplantation to investigate the role of BM-derived microglia, the number of migrating BM-derived cells in normal retinas remains controversial. Xu et al. reported that a majority of retinal microglia/macrophages are replenished from circulating BM-derived cells over 6 months, with very little *in situ* proliferation (i.e. insufficient to account for renewal) (Xu et al., 2007). Kaneko et al. observed bulk BM-derived cell migration to the retina only after the induction of retinal damage (Kaneko et al., 2008). Conversely, systemic irradiation before BM transplantation might ablate resident microglia in the retina, thereby inducing additional BM-derived cell migration. In the current study, we demonstrated that AP20187 efficiently depleted circulating EGFP+ BM-derived cells in *rd7/rd7;Tg/Tg* mice (supplementary material Fig. S2), thus providing better results than BM transplantation, without damaging resident retinal microglial cells. However, we only injected AP20187 after P14 because injection is technically easier at the tail vein after this stage; as such, we could not determine the number of circulating BM-derived cells that migrated to the retina before P14 in *rd7/rd7;Tg/Tg* mice. It is noteworthy that the variance in the number of AF spots in *rd7* mice (Fig. 1E) and *rd7/rd7;Tg/Tg* mice treated with vehicle (Fig. 5J) might not be identical because of an effect of the genetic background and/or the use of ethanol as a vehicle for AP20187 intraperitoneal injection.

In this study, we demonstrated the presence of heterogeneous populations of microglia in the retina that exhibited distinct morphological characteristics, location and distribution in *rd7/rd7;Tg/Tg* mice. Iba-1+ ramified cells have delicate processes, are located in the inner layers of the retina and are considered to be a marker of resident microglia (Sasahara et al., 2008; Joly et al., 2009). CD68 is a cytoplasmic lysosomal antigen and CD68+ amoeboid cells can represent blood-borne macrophages (Milligan et al., 1991). MHC-II+ spindle cells are regarded as antigen-presenting cells and are not present in the normal retina (Zhang et al., 2005). The intermediate filament vimentin is expressed ubiquitously in Müller cells of many mammalian species (Kivelä et al., 1986). However, although different subgroups of microglia might express different markers, we found that, not only do more than 80% of the EGFP+ cells also express Iba-1 in *rd7/rd7;Tg/Tg* mice but, also, these EGFP+ cells are different from Müller cells.

### Distribution of microglia in *rd7/rd7;Tg/Tg* mice

BM-derived cells have been observed along optic nerves and retinal vessels, where activated astrocytes are present, after 24 hours of retinal degeneration induced by N-methyl-N-nitrosourea (MNU) (Kaneko et al., 2008). Similar findings of the presence of BM-derived cells in the ciliary body and in the optic nerve have also been reported in other studies that used BM transplantation (Hou et al., 2006; Xu et al., 2007). In the present study, microglial cells in *rd7* and *rd7/rd7;Tg/Tg* mice were distributed throughout the retina. Additionally, we did not find the pattern that was observed during retinal development (i.e. waves of radial migration along optic nerves and retinal vessels) in *rd7/rd7;Tg/Tg* mice treated with AP20187. One explanation for this finding is that the processes of retinal degeneration in *rd7* and *rd7/rd7;Tg/Tg* mice (with or without AP20187) are subtle and are different from the abrupt degeneration induced by drugs or potential damage during pre-BM-transplantation irradiation. Another explanation is that AP20187 depleted most of the circulating BM-derived cells, resulting in few cells being left in circulation.

In *rd7/rd7;Tg/Tg* mice injected with AP20187, we observed the presence of EGFP+ ramified microglial cells distributed evenly and superficially in the cilioretinal flatmounts. Burnett et al. found no significant EGFP+ cell depletion in brain tissues after AP20187 injection in Mafia mice, and assumed that AP20187 cannot cross the blood-brain barrier (Burnett et al., 2004). Our immunohistochemical findings that Iba-1+ cells were distributed in the GCL and that residual ramified microglial cells remained in the cilioretinal flatmounts after AP20187 injection suggest that these EGFP+ ramified cells are resident microglia and that the dimerizing reagent AP20187 cannot effectively penetrate the blood-retina barrier. However, it should be noted that we could only inject AP20187 through the tail vein or intraperitoneally because AP20187 powder needs to dissolve in ethanol during preparation, which might cause retinal toxicity if injected intravitreally. We attempted retrobulbar injection of AP20187, but injections repeatedly resulted in eyeball ischemia and fibrosis. Additional studies are needed to optimize the dimerizer-treatment protocol to achieve depletion of microglial cells in the CNS and in the retina.

### Inflammation was seen after systemic depletion of circulating BM-derived microglia

Unexpectedly, accelerated retinal degeneration occurred after the observation of the systemic depletion of circulating BM-derived microglia in the ESCS model (Fig. 5). Macrophages and microglial cells become classically activated (M1) or alternatively activated (M2) when affected by Th1- and Th2-derived cytokines, respectively. Several studies have indicated that M1 cells have strong cytotoxic activity, whereas M2 cells promote cell survival and angiogenesis, and suppress destructive immunity (Gratchev et al., 2001; Gratchev et al., 2005; Kzhyshkowska et al., 2006; Kigerl et al., 2009; Seledtsov and Seledtsova, 2012). In the retina, M2 cells secrete neurotrophic factors that promote the survival of neurons during retinal degeneration (Carwile et al., 1998; Harada et al., 2002; Arroba et al., 2011) and vascular repair in a model of ischemic retinopathy (Ritter et al., 2006). Conversely, M1 cells can induce ganglion cell and photoreceptor death by releasing cytotoxic factors (Roque et al., 1999; Zeng et al., 2005; Sivakumar et al., 2011). Inflammatory cytokines related to M1 cells include TNFα, IL-1β and IL-6, whereas cytokines related to M2 cells include IL-10 and TGFβ1. In *rd* mice, the TNFα produced by activated microglia has been found to be neurotoxic (Zeng et al., 2005). In hypoxic neonatal retinas, TNFα and IL-1β also seem to induce retinal ganglion cell death (Sivakumar et al., 2011).

Joly et al. have shown that both resident and BM-derived microglia cooperate to remove dead photoreceptors from retinal lesions (Joly et al., 2009). Therefore, resident microglia initially play a positive role in maintaining the environment of photoreceptors by cleaning up debris between photoreceptors and the RPE. Without the cooperation of circulating BM-derived microglia, resident microglia secrete a monocyte chemotactic protein to try to recruit additional monocytes, as seen in Fig. 7. In addition, resident microglia can proliferate and produce high levels of TNFα and IL-1β, which subsequently lead to the acceleration of retinal degeneration.

In summary, our study demonstrates the sequence of events during the retinal degenerative process in *rd7* mice. Taking advantage of the unique characteristics of Mafia mice, we demonstrated that more than 80% of EGFP+ cells in the retina of *rd7/rd7;Tg/Tg* mice expressed Iba-1, and that resident microglia are still present in the retina because AP20187 does not cross the blood-brain barrier. For this reason, only circulating BM-derived microglia are depleted. After the systemic depletion of circulating BM-derived microglia, the cilioretinal flatmounts from *rd7/rd7;Tg/Tg* mice exhibited characteristics that mimicked those of later stages of retinal degeneration in *rd7* mice. Moreover, ONL analyses confirmed the decrease in the number of nuclei in this region in *rd7/rd7;Tg/Tg* mice. Our finding suggests that the increased number of AF spots is related to resident microglia proliferation, which established a cytokine milieu that was skewed to inflammation by upregulating the expression of the *IL-1β*, *IL-6* and *TNFα* genes, which subsequently accelerated retinal degeneration. Future studies dedicated to the search for therapeutic agents to intervene in the inflammatory processes involved in retinal degeneration should investigate the molecular signals that act between microglial activation and photoreceptor loss.

## MATERIALS AND METHODS

### Mouse strains

All mice were handled in accordance with the Statement for the Use of Animals in Ophthalmic and Vision Research of the Association for Research in Vision and Ophthalmology, and all experiments were approved by the Institutional Animal Care and Use Committee of the Chang Gung Memorial Hospital.

C57BL/6, B6.Cg-*Nr2e3^rd7/rd7^* (*rd7*) and Mafia (Tg/Tg) mice were purchased from the Jackson Laboratory (Bar Harbor, ME) and were housed at a local animal facility under a 12-hour light/12-hour dark cycle. All mice used in this study were in the B6 background. *rd7* mice were outcrossed to Tg/Tg mice, with F_1_ offspring from each of these outcrosses being intercrossed to generate F_2_ mice. The two-generation outcross-intercross series was used to generate *rd7/rd7;Tg/Tg* double homozygotes for the *Nr2e3^rd7/rd7^* mutation and Tg/Tg, as described previously (Haider et al., 2001). The offspring from an intercross of *rd7/rd7;Tg/Tg* mice were used in this study.

### Screening for the transgene in Mafia mice and for the *Nr2e3* gene in *rd7* mice

Genomic DNA was extracted from mouse tails using the QuickExtract kit (Epicentre, Madison, WI). To detect the transgene in Mafia mice, the following EGFP primers were used: 5′-AAGTTCATCTGCACCACCG-3′ (forward) and 5′-TCCTTGAAGAAGATGGTGCG-3′ (reverse). Homozygous *rd7* animals were differentiated from heterozygous and WT controls via PCR analysis of genomic DNA using primers designed for the *Nr2e3* gene. A forward primer located in exon 4 (5′-GTAGCCTCTCCTGCTCTGGCAG-3′) and a reverse primer located in exon 5 (5′-CAGGTTGGAAAACACAGGCAAG-3′) were used to amplify a 339 bp fragment in WT animals and a 239 bp fragment in *rd7* mutants and in heterozygotes harboring the deletion. The fragments were amplified using 5 μl of DNA extract in a 50.0 μl PCR reaction. The cycling conditions were an initial 10 minutes incubation at 94°C, followed by ten cycles of 94°C for 10 seconds, 60°C for 30 seconds and 68°C for 60 seconds, and 20 cycles of 94°C for 10 seconds, 55°C for 30 seconds and 68°C for 60 seconds, and a final extension at 68°C for 7 minutes. PCR products were electrophoresed on 2% agarose gels (Seakem LE, Lonza Rockland, Rockland, ME) and visualized via ethidium bromide staining. The homozygosity of *Tg* mice was determined by calculating the ΔCt using real-time PCR with primers for *EGFP* (test gene) and *ApoB* (reference gene).

### Cilioretinal flatmount and histochemical analyses

Eyes were enucleated and placed in 4% paraformaldehyde for 1 hour at room temperature (RT). The cornea and lens were removed from each eye under a surgical microscope. Whole eyecups were flattened using four to eight radial cuts and mounted with mounting medium (VECTASHIELD, Burlingame, CA). Autofluorescence was detected using standard fluorescence microscopy, as described previously (Wang et al., 2009). Immunostaining was performed as described previously (Wang et al., 2009; Wang et al., 2010).

After fixation as described above, eyes were frozen in optimum cutting temperature compound (Tissue-Tek OCT; Miles Laboratories, Elkhart, IN). Frozen eyes were cryosectioned at a thickness of 10 μm. Blocking was performed using 10% normal blocking serum in PBS with 0.3% Triton X-100 (blocking solution) for 30 minutes at RT. Sections were then incubated with primary antibodies diluted in 4% normal blocking serum in PBS with 0.1% Triton X-100 for 2 hours at RT. The samples were subsequently incubated for 40 minutes at RT with secondary antibodies diluted in PBS with 0.1% Triton X-100. The primary antibodies used were anti-EGFP (1:2000, Novus Biologicals, Littleton, CO), anti-F4/80 (1:100, Abcam, Cambridge, MA), anti-CD68 (1:200, Abcam, Cambridge, MA), anti-ionized calcium-binding adaptor molecule 1 (Iba-1; 1:1000, Wako, Osaka, Japan), anti-major histocompatibility complex (MHC) class II (1:100, BD Pharmingen, San Jose, CA), anti-vimentin (1:200, Sigma-Aldrich, St Louis, MO) and anti-active caspase 3 (1:200, BD Pharmingen, San Jose, CA). The secondary antibodies used were Alexa-Fluor-488- or -555-conjugated antibodies (dilution, 1:1000; Invitrogen, Carlsbad, CA). After being washed three times with PBS, the sections were mounted with mounting medium containing the nuclear dye Hoechst (Invitrogen, Carlsbad, CA) and viewed using a Leica TCS SP5 confocal microscope (Leica Microsystems GmbH, Wetzlar, Germany). Images were merged digitally to assess triple labeling.

The Click-iT 5-ethynyl-2′-deoxyuridine (EdU) labeling kit was used to perform EdU labeling (Invitrogen, Carlsbad, CA). Timed, *rd7/rd7;Tg/Tg* mice were injected with EdU solution (30 μg/g of body weight) continuously for 2 days, and then killed on the third day. Eyes were collected, fixed and processed according to the immunostaining protocol mentioned above. EdU staining was performed according to the protocol provided by the kit.

### Transmission electron microscopy

For TEM, P60 *rd7* and P60 WT mice were first perfused transcardially with 2% glutaraldehyde and 2% paraformaldehyde in phosphate buffer. Subsequently, the eyes of animals were enucleated and placed in 3% glutaraldehyde with 2% paraformaldehyde for 2 hours at 4°C. The cornea was cut and the lens was removed, to allow further fixation for 1 hour. After refixation in 1% osmium tetraoxide for 4 hours at room temperature, the eye samples were prepared as described previously (Yeh et al., 2008). After fixation, the eye samples were washed in phosphate buffer, dehydrated and embedded in Epon 812 epoxy resin. Serial semi-thin sections were obtained along the long axis and stained with toluidine blue. Ultrathin sections from selected areas were collected on 75 mesh copper grids and stained with uranyl acetate and lead citrate, and images were photographed with an Hitachi 7100 transmission electron microscope (Hitachi, Tokyo, Japan) equipped with an AMT digital camera.

### qRT-PCR

Total mRNA was prepared from freshly dissected whole mouse retinas using the TRIzol reagent (Molecular Research Center, Cincinnati, OH). The mRNA was transcribed using SuperScript III Reverse Transcriptase (Invitrogen, Carlsbad, CA) and qRT-PCR assays were performed using the KAPA SYBR® FAST qPCR Kit (Kapa Biosystems, Woburn, MA). Amplified products were run on the Mx3000P™ instrument (Stratagene) in a final reaction volume of 10 μl. The following thermocycling profile was used: denaturation at 95°C for 3 minutes and 40 cycles of 95°C for 3 seconds, 55°C for 20 seconds and 72°C for 1 second. The MxPro software was used to set baselines and CT values according to the guidelines provided by Stratagene. The expression levels of each gene were normalized to the PCR products of β-actin. The following primers were used for qRT-PCR: IL-1β, 5′-TGTGAAATGCCACCTTTTGA-3′ and 5′-CTGCCTGAAGCTCTTGTTGA-3′; IL-6, 5′-TGTGCAATGGCAATTCTGAT-3′ and 5′-CTCTGAAGGACTCTGGCTTTG-3′; IL-10, 5′-TGGCCCAGAAATCAAGGAGC-3′ and 5′-CAGCAGACTCAATACACACT-3′; TNFα, 5′-CCACCACGCTCTTCTGTCTA-3′ and 5′-CACTTGGTGGTTTGCTACGA-3′; TFG-β1, 5′-TTGCTTCAGCTCCACAGAGA-3′ and 5′-TGGTTGTAGAGGGCAAGGAC-3′; MCP-1, 5′-TCTCTTCCTCCACCACTATGCA-3′ and 5′-GGCTGAGACAGCACGTGGAT-3′; β-actin, 5′-TCATGAAGTGTGACGTTGACATCCGT-3′ and 5′-CCTAGAAGCATTTGCGGTGCAGGATG-3′.

### Ablation of BM-derived microglia in transgenic mice

The depletion protocol used here was as described previously (Burnett et al., 2004; Qualls et al., 2006). AP20187 (Ariad Pharmaceuticals, Cambridge, MA) was injected (i.v.) into P14 *rd7/rd7;Tg/Tg* mice once daily at a dose of 10 mg/kg of body weight for 5 consecutive days, for initial cell depletion. This depletion protocol has been shown to cause 80–95% depletion of monocytes, macrophages and DCs in many tissues (Burnett et al., 2004). Depletion can be prolonged by subsequent injection of AP20187 twice weekly at a reduced dose of 1 mg/kg of body weight (Qualls et al., 2006).

### Flow cytometry to determine macrophage and DC depletion

Flow cytometric analysis and sorting were performed on a BD FACSCalibur flow cytometer (BD Biosciences, San Jose, CA) and the data collected were analyzed using the Flowjo software. Peritoneal lavage was performed under anesthesia using 5 ml of PBS and peripheral blood was collected from the tail vein. The preparations were resuspended in tenfold ACK lysis buffer (Lonza Walkersville, Walkersville, MD), spun and processed using flow cytometry. Histograms were graphed from events gated by forward and side scatter (FSC and SSC, respectively) to the region where macrophages and lymphocytes would be found.

### Quantification and statistical analyses

In Fig. 1H, the number of AF spots and rosettes within fields of 200×200 μm (the average of four fields from the central retina and eight fields from the peripheral retina) was determined and processed statistically from montage images of the cilioretinal flatmounts. To compare the EdU-labeled cells in *rd7/rd7;Tg/Tg* mice injected with AP20187 or vehicle, the number of EdU-labeled cells was counted and averaged from 15 serial sections cut around the optic nerve head in each mouse (*n*=3 in each group).

All experimental data were assessed by an operator blinded to the treatment condition. Significance was determined using a paired *t*-test. *P*<0.05 was considered significant. All analyses were performed using the SPSS 15.0 software (SPSS, Chicago, IL).

## Supplementary Material

Supplementary Material
