# Supplementary material for: Origin of fundus hyperautofluorescent spots and their role in retinal degeneration in a mouse model of Goldmann-Favre syndrome
Source: Dis Model Mech. 2013 Jul 4;6(5):1113–22. doi: 10.1242/dmm.012112 (PMC3759331; doi:10.1242/dmm.012112)
Supplement: Supplementary Material [file supp_6_5_1113__index.html]

Origin of fundus hyperautofluorescent spots and their role in retinal degeneration in a mouse model of Goldmann-Favre syndrome — Origin of fundus hyperautofluorescent spots and their role in retinal degeneration in a mouse model of Goldmann-Favre syndrome — Supplementary Material 

# Origin of fundus hyperautofluorescent spots and their role in retinal degeneration in a mouse model of Goldmann-Favre syndrome

## 

**Files in this Data Supplement:**

- **Supplementary Material PDF**
