# Supplementary material for: Origin of fundus hyperautofluorescent spots and their role in retinal degeneration in a mouse model of Goldmann-Favre syndrome
Source: Dis Model Mech. 2013 Jul 4;6(5):1113–22. doi: 10.1242/dmm.012112 (PMC3759331; doi:10.1242/dmm.012112)
Supplement: Supplementary Material [file supp_012112_DMM012112.pdf]

SUPPLEMENTARY FIGURE LEGENDS

Figure S1. Body weight changes after systemic depletion of circulating BM-derived microglia.

A and **B:** After systemic macrophage ablation using AP20187, body weight didn't increase as fast in the AP20187 mice than in the vehicle treated animal.

Figure S2. Systemic Depletion of Circulating BM-derived Microglia

A: Peritoneal cells from an *rd7/rd7;Tg/Tg* mouse were analyzed for EGFP expression using cytometric analysis after treatment with AP20187 (i.v.) for 3 weeks and 6 weeks. Histograms were graphed from events gated by forward and side scatter (FSC and SSC, respectively) to the region where macrophages and lymphocytes would be found. Immunostaining of spleens of *rd7/rd7;Tg/Tg* mice 8 weeks after being injected with vehicle (**B** and **C**) and AP20187 (**E** and **F**) showed more apoptotic cells (brown, active caspase 3; green, TUNEL) in AP20187-injected groups. Nuclei are stained with Hoechst (**C** and **F**). Enlarged spleens were noted in *rd7/rd7;Tg/Tg* mice 8 weeks after injection with AP20187 (**G**) compared with animals injected with vehicle (**D**). Scale bar: (**B, C, E, F**) 20 μm.

Figure S1

A

	AP20187 (No.)	Vehicle (No.)	P Value
BW 2 wks, gm	8.25 ± 1.02 (15)	8.76 ± 1.79 (10)	0.375
BW 4 wks, gm	9.06 ± 0.80 (15)	11.67 ± 2.49 (10)	0.009
BW 6 wks, gm	12.25 ± 1.70 (15)	18.50 ± 1.88 (10)	<0.001
BW 8 wks, gm	16.10 ± 2.18 (15)	22.80 ± 4.17 (10)	0.001
BW 10 wks, gm	17.52 ± 1.75 (14)	24.30 ± 3.36 (10)	<0.001
BW 12 wks, gm	19.15 ± 2.31 (12)	26.24 ± 3.97 (8)	0.001
BW 14 wks, gm	19.47 ± 1.98 (10)	24.80 ± 2.81 (7)	<0.001
BW 16 wks, gm	18.04 ± 1.53 (7)	29.22 ± 1.82 (6)	<0.001
BW 18 wks, gm	18.07 ± 1.18 (5)	27.42 ± 0.86 (6)	<0.001

BW: body weight; No.: number.

B

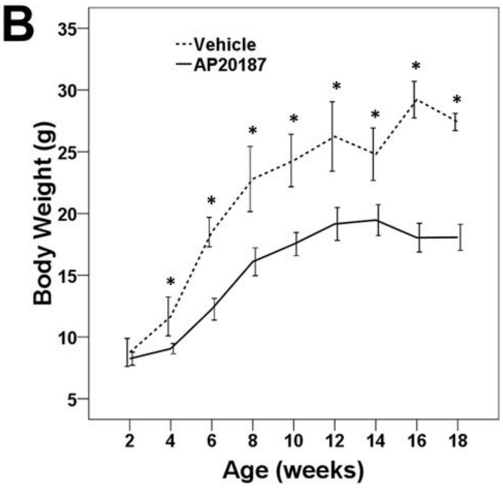

Figure S2

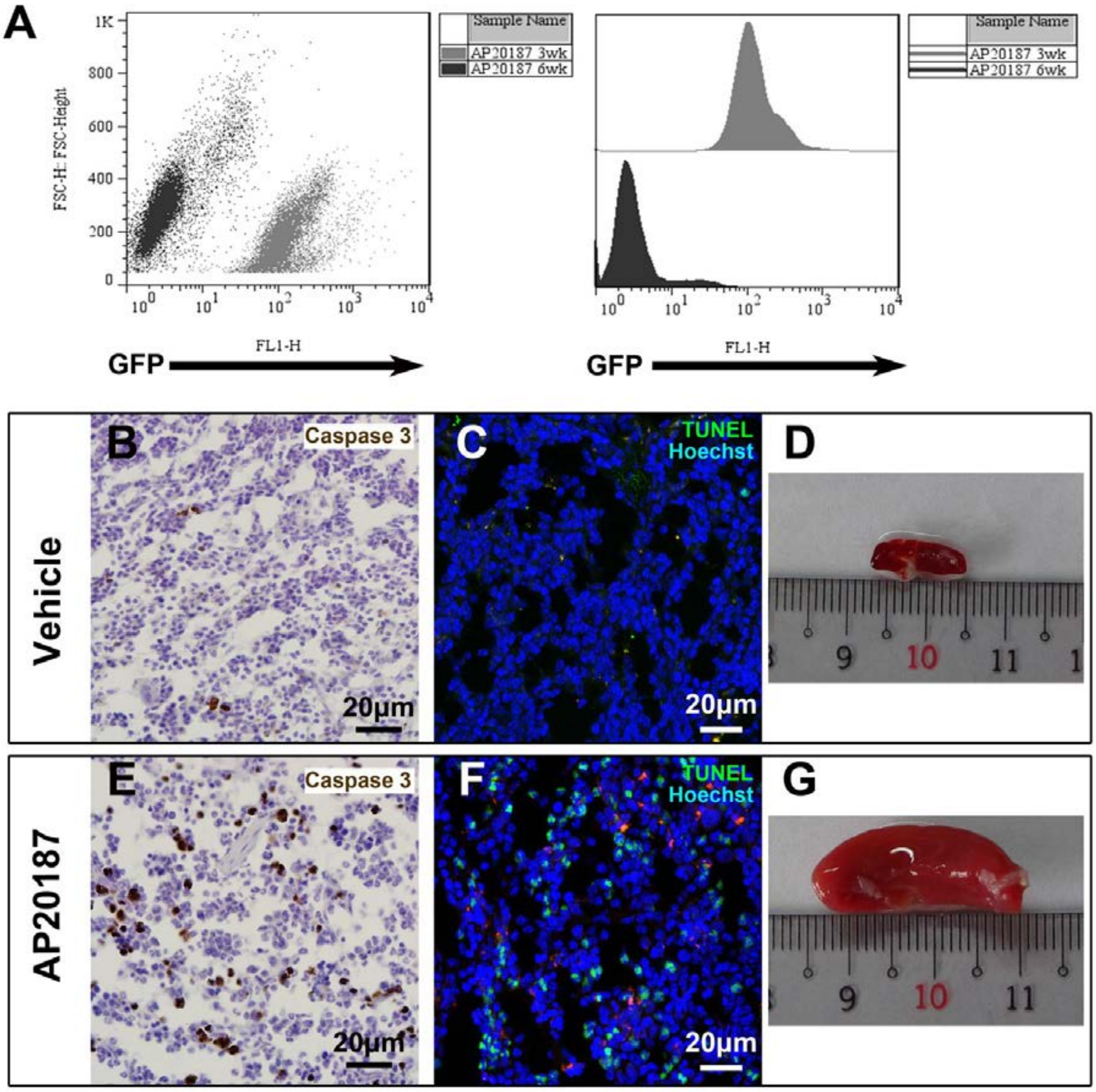
A
Sample Name
AP20187 3wk
AP20187 6wk
FSC-H: FSC-Height
1K
800
600
400
200
0
FL1-H
GFP
Sample Name
AP20187 3wk
AP20187 6wk
FL1-H
GFP
B
Caspase 3
20μm
C
TUNEL
Hoechst
20μm
D
Vehicle
E
Caspase 3
20μm
F
TUNEL
Hoechst
20μm
G
AP20187
